# Supplementary material for: Sour Jujube (Ziziphus jujuba var. spinosa): A Bibliometric Review of Its Bioactive Profile, Health Benefits and Trends in Food and Medicine Applications
Source: Foods. 2024 Feb 20;13(5):636. doi: 10.3390/foods13050636 (PMC10930723; doi:10.3390/foods13050636)
Supplement: Supplementary file 1 [file foods-13-00636-s001.zip › foods-2851387-supplementary.pdf]

Table S1. Flavonoids of different portions in sour jujube

No.	Name	Source	Reference
1	spinosin	seed	[1]
2	6‴-feruloylspinosin	seed	[1]
3	6‴-sinapoylspinosin	seed	[1]
4	6‴-vanilloylspinosin	seed	[2]
5	6‴-*p*-coumaroylspinosin	seed	[1]
6	6‴-*p*-hydroxybenzoylspinosin	seed	[1]
7	6‴-(−)-phaseoylspinosin	seed	[2]
8	6‴-(4⁗-*O*-glc)-vanilloylspinosin	seed	[3]
9	6‴-(4⁗-*O*-glc)-*p*-hydroxybenzoylspinosin	seed	[2]
10	6‴-dihydrophaseoylspinosin	seed	[4]
11	6‴-pyridyloylspinosin	seed	[5]
12	6″,6‴-diferuloylspinosin	seed	[4]
13	meloside A	seed	[1]
14	isovitexin-2″-*O*-*β*-(6-*O*-*E*-feruloyl)-glucopyranoside	seed	[4]
15	6‴-*O*-(3*S*-glc-3-hydroxy-indole-acetyl) spinosin	seed	[6, 7]
16	6‴-*O*-(3*R*-glc-3-hydroxy-indole-acetyl) spinosin	seed	[6, 7]
17	6″-*O*-(3*S*-glc-3-hydroxy-indole-acetyl) spinosin	seed	[5, 6]
18	6″-*O*-(3*R*-glc-3-hydroxy-indole-acetyl) spinosin	seed	[5, 6]
19	6″-*O*-(3*S*-glc-3-hydroxy-indole-acetyl)-6‴-feruloylspinosin	seed	[5, 6]
20	6″-*O*-(3*R*-glc-3-hydroxy-indole-acetyl)-6‴-feruloylspinosin	seed	[5, 6]
21	isospinosin	seed	[1]
22	6‴-feruloylisospinosin	seed	[1]
23	6″,6‴-diferuloylisospinosin	seed	[5]
24	swertisin	seed	[1]
25	isovitexin	seed	[1]
26	vicenin-2	seed	[1]
27	zivulgarin	seed	[1]
28	saponarin	seed	[1]
29	apigenin	seed	[8]
30	kaempferol	seed	[9]
31	quercetin	branch, leaf	[10]
32	quercetin-3-*O*-*β*-(2^{G}-*O*-*β*-xylopyranosyl-6^{G}-O-*α*-rhamnopyranosyl) glucopyranoside	leaf	[11]
33	quercetin-3-*O*-2″-(6″-*p*-coumaroyl)-glucosyl-rhamnoside	leaf	[12]
34	nicotiflorin	fruit, leaf	[12]
35	kaempferol-3-*O*-neohesperidoside	leaf	[12]
36	kaempferol-3-*O*-*α*-L-rhamnopyranosyl-(1→3)-*O*-[*O*-*α*-L-rhamnopyranosyl-(1→6)]-*β*-D-glucopyranoside	seed	[3]
37	camelliaside B	seed	[4]
38	rutin	fruit, leaf, branch	[10, 13, 14]
39	quercetin-3-*O*-*β*-D-glucoside	branch, leaf	[10]
40	kaempferol-3-*O*-robinobioside	fruit	[15]
41	quercetin-3-*O*-robinobioside	leaf	[11]
42	quercetin-3-*O*-*β*-D-galactoside	leaf	[12]
43	quercetin-3-*O*-*α*-L-arabinosyl-(1→2)-*α*-L-rhamnoside	leaf	[8]

44	quercetin-3-*O*-*β*-L-arabinosyl-(1→2)-*α*-L-rhamnoside	leaf	[11]
45	quercetin-3-*O*-*β*-D-xylosyl-(1→2)-*α*-L-rhamnoside	leaf	[11, 13]
46	glycitin	seed	[16]
47	genistin	seed	[16]
48	daidzin	seed	[16]
49	puerarin	seed	[1]
50	nobiletin	seed	[16]
51	spinorhamnoside	seed	[8]
52	hesperidin	seed	[16]
53	clematine	seed	[16]
54	eriodictyol-7-*O*-rutinoside	leaf	[12]
55	naringin	seed	[8]
56	(*R*)-2-hydroxynaringenin	root	[17]
57	pinocembrin	fruit	[18]
58	7,4′-dihydroxy-5-methoxy flavanone	fruit	[18]
59	catechin	seed, root, branch, leaf	[9, 10, 17]
60	5,7,3′,4′-tetramethoxycatechin	fruit	[18]
61	afzelechin	root	[17]
62	gallocatechin	root	[17]
63	epicatechin	seed, root	[9, 17]
64	epiafzelechin	root	[17]
65	epigallocatechin	root	[17]
66	epicatechin 3-*O*-(3-*O*-methylgallate)	leaf	[12]
67	2*R*,3*R*-3,5,7,3′,5′-pentahydroxyflavane	branch, leaf	[10]
68	nervilifordin J	seed	[19]

Table S2. Tetracyclic Triterpenoid Saponins of different portions in sour jujube

No.	Name	Source	Reference
1	jujuboside A	seed	[20]
2	jujuboside B	seed	[20]
3	jujuboside A_1/D	seed	[20]
4	jujuboside B_1	seed	[20]
5	jujuboside C	seed	[20]
6	acetyljujuboside B	seed	[20]
7	*Zizyphus* saponin I	seed, fruit, leaf	[20]
8	*Zizyphus* saponin II	seed, fruit, leaf	[20]
9	*Zizyphus* saponin III	seed	[20]
10	jujuboside I	seed	[20]
11	jujuboside II	seed	[20]
12	jujuboside A_2	seed	[20]
13	jujuboside E	seed	[20]
14	jujuboside III	seed	[20]
15	jujuboside IV	seed	[20]
16	jujubasaponin IV	seed	[21]
17	jujuboside G	seed	[20]
18	jujuboside H	seed	[20]
19	protojujuboside A	seed	[20]
20	protojujuboside B	seed	[20]
21	protojujuboside B_1	seed	[20]

Table S3. Pentacyclic Triterpenoid Saponins of different portions in sour jujube

No.	Name	Source	Reference
	Lupane Type		
1	betulinic acid	seed, fruit, leaf, root, branch	[10, 20]
2	betulin	seed, fruit	[20]
3	betulonic acid	fruit	[20]
4	alphitolic acid	seed, fruit, leaf	[20]
5	lupeol	seed, leaf, root	[20]
6	methyl betulinate	seed	[20]
7	alphitolic acid methyl ester	seed, fruit	[20]
8	2-*O*-protocatechuoylalphitolic acid	root	[20]
9	2α-hydroxypyracrenic acid	root	[20]
10	platanic acid	fruit	[20]
11	3-*O*-*cis*-*p*-coumaroyl alphitolic acid	fruit	[20]
12	3-*O*-*trans*-*p*-coumaroyl alphitolic acid	fruit	[20]
	Ceanothane Type		
13	ceanothic acid	seed, fruit, leaf, root	[20]
14	27-hydroxy ceanothic acid	seed	[20]
15	epiceanothic acid	seed, fruit, leaf	[20]
16	isoceanothic acid	root	[20]
17	24-hydroxyceanothic acid	seed	[5]
18	colubrinic acid	fruit, leaf	[20]
19	3-*O*-protocatechuoyl ceanothic acid	root	[20]
20	ceanothenic acid	fruit, leaf	[20]
	Oleanane Type		
21	oleanolic acid	seed, fruit, leaf	[20]
22	oleanonic acid	fruit	[20]
23	maslinic acid	fruit, leaf	[20]
24	3-*O*-*cis*-*p*-coumaroyl maslinic acid	fruit	[20]
25	3-*O*-*trans*-*p*-coumaroyl maslinic acid	fruit	[20]
26	hydroxyoleanonic acid lactone	fruit	[20]
27	azukisaponin II	seed	[20]
28	*Lathyrus* saponin	seed	[16]
29	zizyphursolic acid	seed	[22]
	Ursane Type		
30	ursolic acid	fruit	[20]
31	ursonic acid	fruit	[20]
32	pomonic acid	seed, fruit	[20]
33	pomolic acid	seed, fruit	[20]
34	pomolic acid 28-methyl ester	seed	[20]
35	corosolic acid	fruit, leaf	[20]
36	cecropiacic acid	fruit	[20]
37	ceanothic acid 28-methyl ester	seed	[20]
38	ceanothic acid 2-methyl ester	seed	[20]

Table S4. Alkaloids of different portions in sour jujube

No.	Name	Source	Reference
1	sanjoinine A	seed	[5]
2	sanjoinine B	seed	[23]
3	sanjoinine F	seed	[23]
4	sanjoinine D	seed	[23]
5	sanjoinine G	seed	[23]
6	sanjoinine G_2	seed	[23]
7	sanjoinenine	seed	[5]
8	adouetine X	seed, root	[24]
9	amphibine D	seed	[5]
10	jubanine C	bark	[25]
11	jubanine E	bark	[26]
12	daechuine S10	root	[17]
13	daechuine S3	seed	[24]
14	mucronine K	seed	[24]
15	paliurine H	seed	[24]
16	nummularine B	seed, root	[24, 25]
17	jubanine F	seed, root	[24, 25]
18	jubanine G	seed	[24]
19	jubanine H	seed	[24]
20	sanjoinine E	seed	[5]
21	sanjoinine Ia	seed	[5]
22	sanjoinine Ib	seed	[5]
23	*N*-methylasimilobine	seed	[5]
24	5-hydroxy-6-methoxynoraporphine	seed	[5]
25	asimilobine	seed	[5]
26	zizyphusine	seed	[5]
27	magnoflorine	seed	[24]
28	lysicamine	seed	[5]
29	sanjoinine K	seed	[24]
30	magnocurarine	seed	[5]
31	lotusine	seed	[5]
32	juzirine	seed	[24]
33	6-(2',3'-dihydroxyl-4'-hydroxymethyl-tetrahydro-furan-1'-yl)-cyclopentene[*c*]-pyrrole-1,3-diol	seed	[3]

Table S5. Other bioactive compounds of different portions in sour jujube

No.	Name	Source	Reference
1	pseudolaroside B	seed	[5]
2	chlorogenic acid	fruit	[27]
3	3′,5′-di-*C*-*β*-D-glucosylphloretin	leaf	[12]
4	4-hydroxy-2-methoxyphenyl-6-*O*- syringoyl-*β*-D-glucopyranoside	root	[17]
5	2-methoxyhydroquinone-4-*O*-[6-*O*-(4-*O*-*α*-L-rhamnopyranosyl)- syringyl]-*β*-D-glucopyranoside	root	[17]
6	jujuphenoside	seed	[16]
7	stigmasterol	root	[14]
8	stigmast-5-en-3*β*,7*α*-diol	fruit	[18]
9	stigmast-5,22-ene-3*β*,7*α*-diol	fruit	[18]
10	3*S*-1-*N*-*β*-D-glc-2-oxo-3-hydroxy-indole-3-acetic acid	seed	[6]
11	3*R*-1-*N*-*β*-D-glc-2-oxo-3-hydroxy-indole-3-acetic acid	seed	[6]
12	(*R*)-3-phenyl lactic acid methyl ester	branch, leaf	[10]
13	(*S*)-3-(3-indolyl) lactic acid methyl ester	branch, leaf	[10]
14	salicylic acid	fruit	[14]
15	protocatechuic acid	seed	[28]
16	ferulic acid	seed	[29]
17	caffeic acid	fruit	[27]
18	dihydrophaseic acid 3-*O*-*β*-D-glucopyranoside	seed	[30]
19	alismoxide	seed	[16]
20	malic acid	fruit	[14]
21	4-ethyl-2-hydroxysuccinate	fruit	[14]

Reference

[1] Niu, C.Y.; Wu, C.S.; Sheng, Y.X.; Zhang, J.L. Identification and characterization of flavonoids from *semen zizyphi spinosae* by high-performance liquid chromatography/linear ion trap FTICR hybrid mass spectrometry. *J. Asian Nat. Prod. Res.* **2010**, 12, 300-312.

[2] Zhu, X.; Liu, X.; Pei, K.; Duan, Y.; Zhu, H.; Ma, J.; Xu, Y.; Wu, Z.; Zhou, Q.; Cai, B. Development of an analytical strategy to identify and classify the global chemical constituents of *Ziziphi Spinosae Semen* by using UHPLC with quadrupole time-of-flight mass spectrometry combined with multiple data-processing approaches. *J. Sep. Sci.* **2018**, 41.

[3] Xie, Y.; Xu, Z.; Wang, H.; Kano, Y.; Yuan, D. A novel spinosin derivative from *Semen Ziziphi Spinosae*. *J. Asian Nat. Prod. Res.* **2011**, 13, 1151-1157.

[4] Zhang, L.; Xu, Z.L.; Wu, C.F.; Yang, J.Y.; Kano, Y.; Yuan, D. Two new flavonoid glycosides from *Semen Ziziphi Spinosae*. *J. Asian Nat. Prod. Res.* **2012**, 14, 121-128.

[5] Zhang, F.X.; Li, M.; Qiao, L.R.; Yao, Z.H.; Li, C.; Shen, X.Y.; Wang, Y.; Yu, K.; Yao, X.S.; Dai, Y. Rapid characterization of *Ziziphi Spinosae Semen* by UPLC/Qtof MS with novel informatics platform and its application in evaluation of two seeds from *Ziziphus* species. *J. Pharm. Biomed. Anal.* **2016**, 122, 59-80.

[6] Li, M.; Wang, Y.; Tsoi, B.; Jin, X.J.; He, R.R.; Yao, X.J.; Dai, Y.; Kurihara, H.; Yao, X.S. Indoleacetic acid derivatives from the seeds of *Ziziphus jujuba* var. *spinosa*. *Fitoterapia* **2014**, 99, 48-55.

[7] Wu, Y.; Zhang, J.; Chen, M.; Yu, B.W.; Wang, D.Y.; Liu, J.G.; Hu, Y.L. C-glucosyl flavones from *Ziziphus jujuba* var. *spinosa*. *Chem. Nat. Compd.* **2015**, 51, 247-251.

[8] Guo, S.; Duan, J.A.; Qian, D.W.; Tang, Y.P. Chemical constituents of *Ziziphus* plants: Research advances. *J. Int. Pharm. Res.* **2013**, 40, 702-710.

[9] Mao, Y.N.; Wu, W.Q.; Kang, Y.; Wang, F.F.; Liu, Y. Rapid analysis of chemical components in *Zizyphi Spinosae Semen* by UHPLC-LTQ-Orbitrap-MS. *China J. Chin. Mater. Med.* **2018**, 43, 4884-4891.

[10]Zhang, Q.Q.; Wen, Q.I.; Wang, W.N.; Li, M.X.; Dang, F.; Liu, X.Q. Isolation and identification of chemical constituents from *Ziziphi Spinosae* Branch and Folium. *J. Shenyang Pharm. Univ.* **2013**, 30, 917.

[11]Zhang, R.T.; Chen, J.H.; Shi, Q.; Li, Z.Y.; Peng, Z.Y.; Zheng, L.; Wang, X.R. Quality control method for commercially available wild Jujube leaf tea based on HPLC characteristic fingerprint analysis of flavonoid compounds. *J. Sep. Sci.* **2014**, 37, 45-52.

[12]Yan, Y.; Cai, F.U.; Du, C.H. Research progress on nutrient composition, health functions and product development of *Ziziphi Spinosae* Folium. *Sci. Technol. Food Ind.* **2018**, 39, 330.

[13]Guo, S.; Duan, J.a.; Tang, Y.P.; Qian, Y.F.; Zhao, J.L.; Qian, D.W.; Su, S.L.; Shang, E.X. Simultaneous qualitative and quantitative analysis of triterpenic acids, saponins and flavonoids in the leaves of two *Ziziphus* species by HPLC-PDA-MS/ELSD. *J. Pharm. Biomed. Anal.* **2011**, 56, 264-270.

[14]Guo, S.; Duan, J.A.; Zhao, J.L.; Qian, Y.F.; Qian, D.W. Resource chemical constituents from sarcocarp of *Ziziphus jujuba* var. *spinosa*. *Chin. Tradit. Herb. Drugs* **2012**, 43, 1905-1909.

[15]Guo, S.; Duan, J.A.; Shen, J.; Xia, N.; Tang, Y.P.; Meng, F.W.; Shang, E.X.; Qian, D.W. Binary detector fingerprints analysis of *Ziziphus jujuba* and *Ziziphus jujuba* var. *spinosa* by

HPLC-DAD-ELSD coupled with chemometric method. *J. Liq. Chromatogr. Relat. Technol.* **2011**, 34, 2048-2062.

[16]Li, L.M.; Liao, X.; Peng, S.L.; Ding, L.S. Chemical constituents from the seeds of *Ziziphus jujuba* var. *spinosa* (Bunge) Hu. *J. Integr. Plant Biol.* **2005**, 47, 494-498.

[17]Meng, Y.; Zhang, Y.; Jiang, H.; Bao, Y.; Wu, Y.; Sun, L.; Yu, C.; Huang, Y.; Li, Y. Chemical constituents from the roots of *Zizyphus jujuba* Mill. var. *spinosa*. *Biochem. Syst. Ecol.* **2013**, 50, 182-186.

[18]Wu, Y.; Chen, M.; Du, M.B.; Yue, C.H.; Li, Y.Y.; Zhu, M.; Liu, C.; Wang, D.Y.; Liu, J.G.; Hu, Y.L. Chemical constituents from the fruit of *Zizyphus jujuba* Mill. var. *spinosa*. *Biochem. Syst. Ecol.* **2014**, 57, 6-10.

[19]Yan, Y.; Li, Q.; Du, C.H.; Jia, J.P.; Qin, X.M. Investigation of the potentially effective components of *Semen Ziziphi Spinosae* based on "*in vitro* to *in vivo*" translation approach. *Acta Pharm. Sin.* **2017**, 52, 283-290.

[20]Du, C.H.; Cui, X.F.; Pei, X.P.; Yan, Y.; Qin, X.M. Research progress on *Ziziphi Spinosae Semen* saponins and its biological action on nervous system. *Chin. Tradit. Herb. Drugs* **2019**, 50, 1258-1268.

[21]Fu, Q.; Yuan, H.M.; Chen, J.; Shi, J.Y. Dammarane-type saponins from *Ziziphus jujube* and their inhibitory effects against TNF-α release in LPS-induced RAW 246.7 macrophages. *Phytochem. Lett.* **2016**, 16, 169-173.

[22]Yang, B.; Yang, H.; Chen, F.; Hua, Y.; Jiang, Y. Phytochemical analyses of *Ziziphus jujuba* Mill. var. *spinosa* seed by ultrahigh performance liquid chromatography-tandem mass spectrometry and gas chromatography-mass spectrometry. *Analyst* **2013**, 138, 6881-6888.

[23]Hwang, K.H.; Han, Y.N.; Han, B.H. Inhibition of calmodulin-dependent Calcium-ATPase and phosphodiesterase by various cyclopeptides and peptide alkaloids from the *Zizyphus* species. *Arch. Pharmacal Res.* **2001**, 24, 202-206.

[24]Kang, K.B.; Jang, D.S.; Kim, J.; Sung, S. UHPLC-ESI-qTOF-MS analysis of cyclopeptide alkaloids in the seeds of *Ziziphus jujuba* var. *spinosa*. *Mass Spectrom. Lett.* **2016**, 7, 45-49.

[25]Shergis, J.L.; Ni, X.; Sarris, J.; Zhang, A.L.; Guo, X.; Xue, C.C.; Lu, C.; Hugel, H. *Ziziphus spinosa* seeds for insomnia: A review of chemistry and psychopharmacology. *Phytomedicine* **2017**, 34, 38-43.

[26]Pandey, M.B.; Singh, A.K.; Singh, J.P.; Singh, V.P.; Pandey, V.B. Three new cyclopeptide alkaloids from *Zizyphus* species. *J. Asian Nat. Prod. Res.* **2008**, 10, 709-713.

[27]Hudina, M.; Liu, M.; Veberic, R.; Stampar, F.; Colaric, M. Phenolic compounds in the fruit of different varieties of Chinese jujube (*Ziziphus jujuba* Mill.). *J. Hortic. Sci. Biotechnol.* **2008**, 83, 305-308.

[28]Wang, D.; Li, Q.; Liu, R.; Xu, H.; Yin, Y.; Wang, Y.; Wang, H.; Bi, K. Quality control of *Semen Ziziphi Spinosae* standard decoction based on determination of multi-components using TOF-MS/MS and UPLC-PDA technology. *J. Pharm. Anal.* **2019**, 9, 406-413.

[29]He, B.; Li, Q.; Jia, Y.; Zhao, L.; Xiao, F.; Lv, C.; Xu, H.; Chen, X.; Bi, K. A UFLC-MS/MS method for simultaneous quantitation of spinosin, mangiferin and ferulic acid in rat plasma: application to a comparative pharmacokinetic study in normal and insomnic rats. *J. Mass Spectrom.* **2012**, 47, 1333-1340.

[30]Lee, S.Y.; Kim, J.S.; Lee, J.H.; Kim, Y.S.; Kang, S.S. A new saponin from the seeds of *Zizyphus jujuba* var. *spinosa*. *Bull. Korean Chem. Soc.* **2013**, 34, 657-660.
